# Toxicity of lead: A review with recent updates

**DOI:** 10.2478/v10102-012-0009-2

**Published:** 2012-06

**Authors:** Gagan Flora, Deepesh Gupta, Archana Tiwari

**Affiliations:** School of Biotechnology, Rajiv Gandhi Proudyogiki Vishwavidyalaya, Bhopal, M.P. INDIA

**Keywords:** antioxidants, reactive oxygen species, lead toxicity

## Abstract

Lead poisoning has been recognized as a major public health risk, particularly in developing countries. Though various occupational and public health measures have been undertaken in order to control lead exposure, cases of lead poisoning are still reported. Exposure to lead produces various deleterious effects on the hematopoietic, renal, reproductive and central nervous system, mainly through increased oxidative stress. These alterations play a prominent role in disease manifestations. Modulation of cellular thiols for protection against reactive oxygen species (ROS) has been used as a therapeutic strategy against lead poisoning. N-acetylcysteine, α-lipoic acid, vitamin E, quercetin and a few herbal extracts show prophylaxis against the majority of lead mediated injury in both *in vitro* and *in vivo* studies. This review provides a comprehensive account of recent updates describing health effects of lead exposure, relevant biomarkers and mechanisms involved in lead toxicity. It also updates the readers about recent advances in chelation therapy and newer therapeutic strategies, like nanoencapsulation, to treat lead induced toxic manifestations.

**LIST OF ABBREVIATIONS**
Pb: Lead
ROS: Reactive oxygen species
GSH: Glutathione
GSSG: glutathione disulfide
ALAD: δ-aminolevulinic acid dehydratase
ALAS: δ-aminolevulinic acid synthetase
ZPP: zinc protoporphyrin
GPx: glutathione peroxidase
CAT: Catalase
SOD: Superoxide dismutase
DMSA: 2,3-dimercaptosuccinic acid
MiADMSA: Monoisoamy dimercaptosuccinic acid
BBB: Blood-brain barrier
PPR: Paired-Pulse Reactions
EPSP: Excitatory postsynaptic potential
PS: Population spike

## Introduction

Lead (Pb) is ubiquitous and one of the earliest metals discovered by the human race. Unique properties of lead, like softness, high malleability, ductility, low melting point and resistance to corrosion, have resulted in its widespread usage in different industries like automobiles, paint, ceramics, plastics, *etc*. This in turn has led to a manifold rise in the occurrence of free lead in biological systems and the inert environment.

Lead is regarded as a potent occupational toxin and its toxicological manifestations are well known. The non biodegradable nature of lead is the prime reason for its prolonged persistence in the environment. Human exposure to lead occurs through various sources like leaded gasoline, industrial processes such as lead smelting and coal combustion, lead-based paints, lead containing pipes or lead-based solder in water supply systems, battery recycling, grids and bearings, *etc*. Although lead toxicity is a highly explored and comprehensively published topic, complete control and prevention over lead exposure is still far from being achieved. There is no such level of lead that appears to be necessary or beneficial to the body and no “safe” level of exposure to lead has been found. Lead toxicity is a particularly insidious hazard with the potential of causing irreversible health effects. It is known to interfere with a number of body functions and it is primarily affecting the central nervous, hematopoietic, hepatic and renal system producing serious disorders (Kalia & Flora, 2005). Acute toxicity is related to occupational exposure and is quite uncommon. Chronic toxicity on the other hand is much more common and occurs at blood lead levels of about 40–60 ug/dL. It can be much more severe if not treated in time and is characterized by persistent vomiting, encephalopathy, lethargy, delirium, convulsions and coma (Flora *et al.,* 2006; Pearce, 2007).

## Effect on the Nervous System

Compared to other organ systems, the nervous system appears to be the most sensitive and chief target for lead induced toxicity (Cory-Slechta, 1996). Both the central nervous system and the peripheral nervous system become affected on lead exposure. The effects on the peripheral nervous system are more pronounced in adults while the central nervous system is more prominently affected in children (Brent, 2006; Bellinger, 2004). Encephalopathy (a progressive degeneration of certain parts of the brain) is a direct consequence of lead exposure and the major symptoms include dullness, irritability, poor attention span, headache, muscular tremor, loss of memory and hallucinations. More severe manifestations occur at very high exposures and include delirium, lack of coordination, convulsions, paralysis, coma and ataxia (Flora *et al.,* 2006). Fetuses and young children are especially vulnerable to the neurological effects of lead as the developing nervous system absorbs a higher fraction of lead. The proportion of systemically circulating lead gaining access to the brain of children is significantly higher as compared to adults (Needleman *et al.,* 2004). Children may appear inattentive, hyperactive and irritable even at low lead exposure. Children with greater lead levels may be affected with delayed growth, decreased intelligence, short-term memory and hearing loss. At higher levels, lead can cause permanent brain damage and even death (Cleveland *et al.,* 2008). There is evidence suggesting that low level lead exposure significantly affects IQs along with behavior, concentration ability and attentiveness of the child. Repercussions of lead exposure on the peripheral nervous system have also been observed in the form of peripheral neuropathy, involving reduced motor activity due to loss of myelin sheath which insulates the nerves, thus seriously impairing the transduction of nerve impulses, causing muscular weakness, especially of the exterior muscles, fatigue and lack of muscular co-ordination (Sanders *et al.*, 2009).

## Effect on the Hematopoietic System

Lead directly affects the hematopoietic system through restraining the synthesis of hemoglobin by inhibiting various key enzymes involved in the heme synthesis pathway. It also reduces the life span of circulating erythrocytes by increasing the fragility of cell membranes. The combined aftermath of these two processes leads to anemia (Guidotti *et al.*, 2008; Cornelis., 2005). Anemia caused on account of lead poisoning can be of two types: ***hemolytic anemia***, which is associated with acute high-level lead exposure, and ***frank anemia***, which is caused only when the blood lead level is significantly elevated for prolonged periods (Vij, 2009).

**Table 1:** Types of lead poisoning.

	Exposure	Lead levels (µg/dl)	Clinical symptoms
**Acute poisoning**	Intense exposure of short duration	100–120	Muscle pain, fatigue, abdominal pain, headache, vomiting, seizures and coma
**Chronic poisoning**	Repeated low-level exposure over a prolonged period	40–60	Persistent vomiting, encephalopathy, lethargy, delirium, convulsions and coma

Lead significantly affects the heme synthesis pathway in a dose dependent manner by downregulating three key enzymes involved in the synthesis of heme. ***δ-aminolevulinic acid dehydratase (ALAD)***, a cytosolic enzyme that catalyzes the formation of porphobilinogen from δ-aminolevulinic acid (ALA), ***aminolevulinic acid synthetase (ALAS)***, a mitochondrial enzyme that catalyzes the formation of aminolevulinic acid (ALA), and finally, the mitochondrial enzyme ***ferrochelatase*** that catalyzes the insertion of iron into protoporphyrin to form heme (Piomelli, 2002). The initial and final steps of heme synthesis take place in the mitochondria, whereas the intermediate steps take place in the cytoplasm.

Lead inhibits the three aforementioned vital enzymes of this pathway but its effect on ALAD is more profound and its inhibition has been used clinically to gauge the degree of lead poisoning. Inhibition of ALAD results in the accumulation of aminolevulinic acid, detectable in the plasma and urine even at blood lead levels of less than 10 µg/dl. Although ALAD inhibition is first noted at blood lead levels of 10–20 µg/dl, heme biosynthesis does not decrease until the activity of ALAD is inhibited by 80–90%, which occurs at a much higher blood lead concentration of about 55 µg/dl (Ahamed *et al.*, 2005). Inhibition of ferrochelatase results in increased excretion of coproporphyrin in urine and accumulation of protoporphyrin in erythrocytes (EP). Moreover, inhibition of this enzyme results in the substitution of iron by zinc in the porphyrin ring forming zinc protoporphyrin (ZPP). The concentration of ZPP thus gets increased, which can also be used as an indicator to monitor the level of lead exposure (Jangid *et al.*, 2012). Thus, the collective inhibition of these three key enzymes blocks the heme production via the heme synthesis pathway. The mechanism responsible for shortening the life cycle of erythrocytes is not well understood. One of the earliest observed hematological effects of lead revealed basophilic stipplings of red blood cells (presence of dense material in red blood cells), which is also a potential biomarker for the detection of lead poisoning. These aggregates are degradation products of ribonucleic acid (Patrick, 2006).

## Renal Effects

Renal dysfunction occurs mostly at high levels of lead exposure (>60 µg/dL) but damage at lower levels has also been reported (∼10 µg/dL) (Grant, 2008). Renal functional abnormality can be of two types: acute nephropathy and chronic nephropathy. Acute nephropathy is characterized functionally by an impaired tubular transport mechanism and morphologically by the appearance of degenerative changes in the tubular epithelium along with the occurrence of nuclear inclusion bodies containing lead protein complexes. It does not cause protein to appear in the urine but can give rise to abnormal excretion of glucose, phosphates and amino acids, a combination referred to as ***Fanconi's syndrome***. Chronic nephropathy on the other hand, is much more severe and can lead to irreversible functional and morphological changes. It is characterized by glomerular and tubulointerstitial changes, resulting in renal breakdown, hypertension and hyperuricemia (Rastogi, 2008).

## Cardiovascular Effects

Both chronic and acute lead poisoning causes cardiac and vascular damage with potentially lethal consequences including hypertension and cardiovascular disease (Navas-Acien *et al.*, 2007). Low level lead exposure can contribute to hypertension in both animals and humans (ATSDR, 2005). Other major disorders include ischemic coronary heart disease, cerebrovascular accidents and peripheral vascular disease. Although evidence of causal relationship of lead exposure and hypertension was reported, it applies only in cases of cardiovascular outcomes of lead toxicity (Navas-Acien *et al.,* 2007).

## Reproductive Health Effects

Lead causes a number of adverse effects on the reproductive system in both men and women. Common effects seen in men include: reduced libido, abnormal spermatogenesis (reduced motility and number), chromosomal damage, infertility, abnormal prostatic function and changes in serum testosterone. Women on the other hand, are more susceptible to infertility, miscarriage, premature membrane rupture, pre-eclampsia, pregnancy hypertension and premature delivery (Flora *et al.*, 2011). Moreover, during the gestation period, direct influence of lead on the developmental stages of the fetus has also been reported (Saleh *et al.*, 2009).

## Effect on Bone

The primary site of lead storage in the human body are bones (Renner, 2010; Silbergeld *et al.,* 1993). There are two compartments in bones where lead is believed to be stored. The exchangeable pool present at the surface of bone and the non-exchangeable pool located deeper in the cortical bone. Lead can enter into plasma at ease from the exchangeable pool but can leave the non-exchangeable pool and move to the surface only when bone is actively being re-absorbed (Patrick, 2006). Stable lead isotope methodology showed that bones contibute around 40–70% of lead released into blood in adults. In adults, 85–95% of the lead is stored in bones, in contrast to 70% in children, resulting in higher concentration of lead in soft tissues in children. The storage and the mobilization of lead in bones depends on several factors, like dose/rate of lead exposure, age, pregnancy, gestation and race.

## Mechanism of toxicity

Lead is probably the most extensively studied heavy metal. Studies carried out in this field have reported the presence of various cellular, intracellular and molecular mechanisms behind the toxicological manifestations caused by lead in the body.

### Oxidative stress

**Figure 1:**
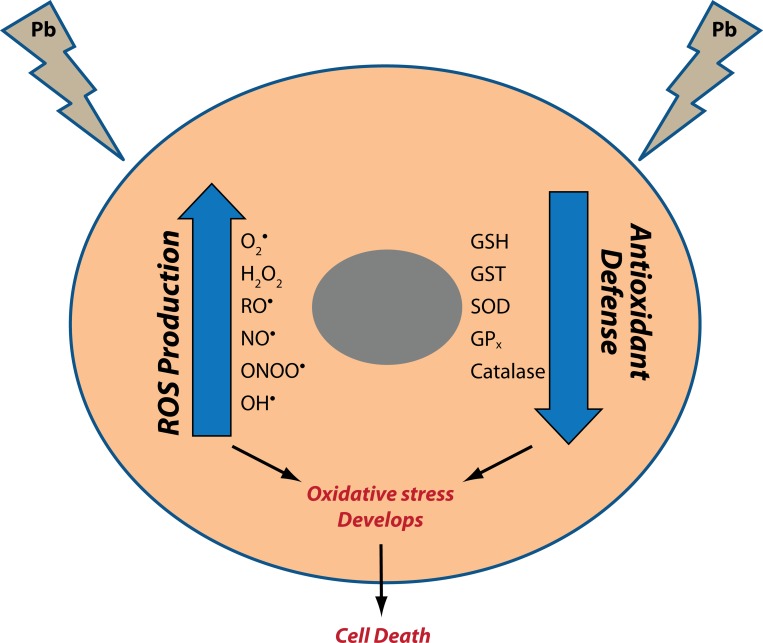
Mechanism underlying the development of oxidative stress in a cell on lead exposure.

Oxidative stress represents an imbalance between the production of free radicals and the biological system's ability to readily detoxify the reactive intermediates or to repair the resulting damage (Flora, 2011). It has been reported as a major mechanism of lead induced toxicity. Under the influence of lead, onset of oxidative stress occurs on account of two different pathways operative simultaneously; first comes the ***generation of ROS,*** like hydroperoxides (HO_2_^•^), singlet oxygen and hydrogen peroxide (H_2_O_2_), and second, ***the antioxidant reserves become depleted*** (Flora *et al.,* 2002) (Figure 1).

**Figure 2:**
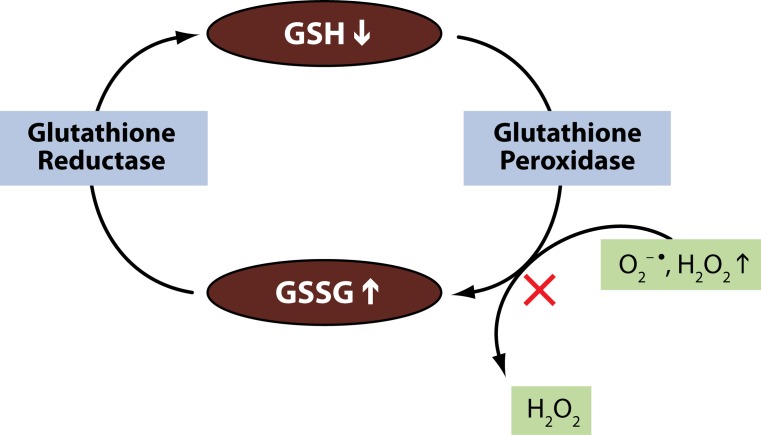
Effect of lead on GSH metabolism.

The antioxidant defenses of the body come into play to nullify the generated ROS. The most important antioxidant found in cells is g***lutathione (GSH)***. It is a tripeptide having sulfhydryl groups and is found in mammalian tissues in millimolar concentrations. It is an important antioxidant for quenching free radicals (Mates, 2000). Glutathione exists in both reduced (GSH) and oxidized form (GSSG). The reduced state of glutathione donates reducing equivalents (H^+^ + e^–^) from its thiol groups present in cysteine residues to ROS and makes them stable. After donating the electron, it readily combines with another molecule of glutathione and forms ***glutathione disulfide (GSSG)*** in the presence of the enzyme ***glutathione peroxidase (GP*** *_**X**_**)***. GSH can be regenerated from GSSG by the enzyme g***lutathione reductase (GR)*** (Figure 2). Under normal conditions, 90% of the total glutathione content exists in reduced form (GSH) and around 10% is in the oxidized form (GSSG). Under conditions of oxidative stress, the concentration of GSSG is much higher than that of GSH.

Lead shows electron sharing capability that results in the formation of covalent attachments. These attachments are formed between the lead moiety and the sulfhydryl groups present in antioxidant enzymes, which are the most susceptible targets for lead and which eventually get inactivated. Lead inactivates glutathione by binding to sulfhydryl groups present in it. This results in synthesis of GSH from cysteine via the γ-glutamyl cycle, which is usually not effective in replenishing the supply of GSH (Hultberg *et al.,* 2004). Similarly, lead inactivates enzymes like δ-amino levulinic acid dehydratase (ALAD), glutathione reductase (GR), glutathione peroxidase (GP_X_) and glutathione-S-transferase, which further depresses the glutathione levels (Ahamed & Siddiqui, 2007).

A few other notable antioxidant enzymes that are rendered inactive by lead include ***super oxide dismutase*** (SOD) and ***catalase*** (CAT). Decrease in SOD concentration reduces the disposal of superoxide radical, whereas reduction in CAT impairs scavenging of superoxide radical (O_2_^– •^). Apart from targeting the sulfhydryl groups, lead can also replace the zinc ions that serve as important co-factors for these antioxidant enzymes and inactivates them (Flora *et al.,* 2007).

Lipid peroxidation is another biomarker of oxidative stress and is one of the most investigated consequences of ROS on lipid membranes. The generated free radical captures electrons from the lipids present inside the cell membranes and damages the cell. Apart from lipid peroxidation, lead also causes hemoglobin oxidation, which directly causes RBC hemolysis. This occurs due to inhibition of ALAD, which results in an increased concentration of substrate ALA in both blood and urine. These elevated ALA levels generate hydrogen peroxide and superoxide radical and also interact with oxyhemoglobin, resulting in the generation of hydroxyl radicals (Patrick, 2006). Progression of all the above mentioned mechanisms makes the cell extremely vulnerable to oxidative stress and may lead to cell death.

### Ionic mechanism of lead toxicity

Ionic mechanism of action for lead mainly arises due to its ability to substitute other bivalent cations like Ca^2+^, Mg^2+^, Fe^2+^ and monovalent cations like Na^+^ (though bivalent cations are more readily substituted), affecting various fundamental biological processes of the body (Lidsky & Schneider, 2003). Significant effects have been found on various fundamental cellular processes like intra and intercellular signaling, cell adhesion, protein folding and maturation, apoptosis, ionic transportation, enzyme regulation, release of neurotransmitters, *etc.* (Garza *et al.,* 2006). The ionic mechanism contributes principally to neurological deficits, as lead, after replacing calcium ions, becomes competent to cross the blood-brain barrier (BBB) at an appreciable rate. After crossing the BBB, lead accumulates in astroglial cells (containing lead binding proteins). Toxic effects of lead are more pronounced in the developing nervous system comprising immature astroglial cells that lack lead binding proteins. Lead easily damages the immature astroglial cells and obstructs the formation of myelin sheath, both factors involved in the development of BBB.

Lead, even in picomolar concentration, can replace calcium, thereby affecting key neurotransmitters like protein kinase C, which regulates long term neural excitation and memory storage. It also affects the sodium ion concentration, which is responsible for numerous vital biological activities like generation of action potentials in the excitatory tissues for the purpose of cell to cell communication, uptake of neurotransmitters (choline, dopamine and GABA) and regulation of uptake and retention of calcium by synaptosomes. This interaction between lead and sodium seriously impairs the normal functioning of the aforementioned sodium dependent processes (Bressler *et al.,* 1999).

## Prevention of lead induced toxicity

Preventive measures are preferred over the treatment regimens, considering the toxic effects of lead. This is due to the fact that once lead enters the body, it is almost impossible to remove it completely or to reverse its damaging effects on the body. Guidotti and Ragain (2007) suggested a three-way measure as preliminary preventive approach towards lead toxicity. It includes *Individual intervention*, *Preventive medicine strategy* and *Public health strategy*.

Preventive medicine strategy mainly aims at screening the blood levels of children that are at a high risk of lead exposure. If lead is detected in blood, medical intervention is carried out with the aim to control undesirable outcomes of poisoning and prevent further accumulation of lead.

Public health strategy has a much larger sphere of influence and acts at a population level with a target to reduce the risk of lead exposure in habitable regions. Various preventive strategies have been suggested by the public health services for controlling lead. The most important of them include: prohibition of setting up industries dealing with lead close to habitable areas and completely banning the use of lead where appropriate replacement is available.

Apart from the above mentioned preliminary strategies, nutrition also plays an important role in prevention of lead induced toxicity. Studies have shown that uptake of certain nutrients like mineral elements, flavonoids and vitamins can provide protection from the environmental lead as well as from the lead already present in the body. These nutrients play a pivotal role in restoring the imbalanced prooxidant/oxidant ratio that arises due to oxidative stress. Although the mechanism by which these nutrients restore the delicate prooxidant/oxidant ratio is still unclear, significant data are available suggesting a protective role of nutrients against lead poisoning (Hsu & Guo, 2002).

## Role of antioxidants in protecting lead induced oxidative stress

**Figure 3:**
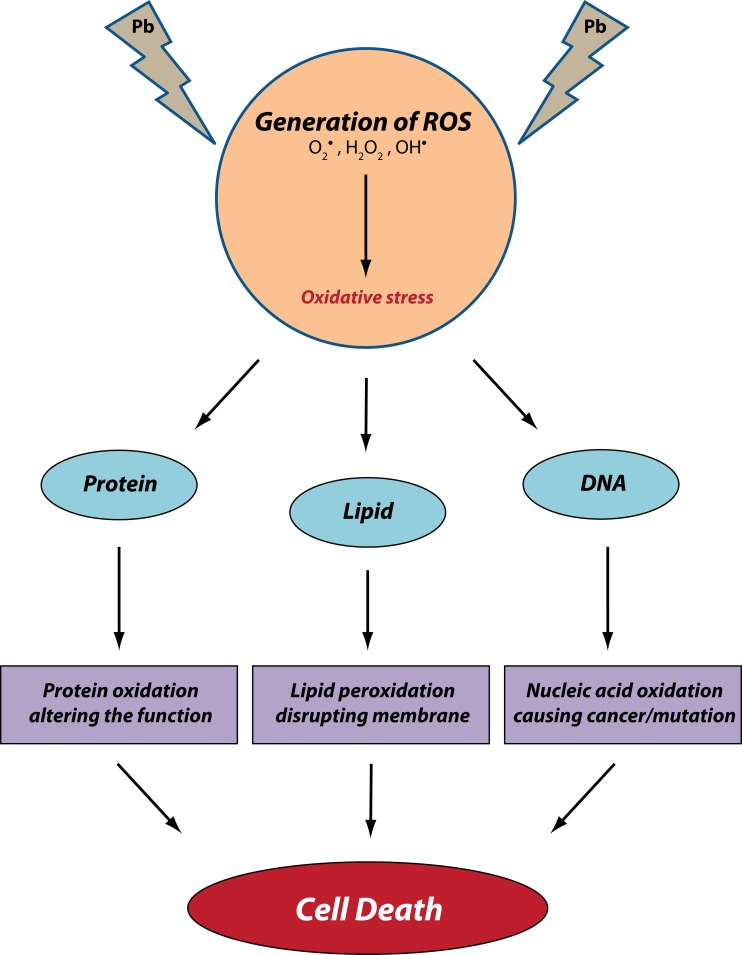
Possible mechanism and targets for lead-induced oxidative stress.

Lead stimulated oxidative stress is a state that involves the generation of free radicals beyond the permissible limits, depleting at the same time the antioxidant reserves and thus hampering the ability of the biological system to reverse the resulting effects. Free radicals generation starts a chain reaction that results in lipid peroxidation, disruption of cell membrane, protein oxidation and oxidation of nucleic acids like DNA and RNA leading to cancer (Gurer & Ercal, 2000) (Figure 3). Research findings have suggested that administration of various antioxidants can prevent or subdue various toxic effects of lead and generation of oxidative stress in particular.

An *antioxidant* is a substance which, when present at a low concentration as compared to that of the oxidizable substrate, can prevent the oxidation of that substrate. Generally, an antioxidant can prevent lead toxicity in three ways (Garcia & Gonzalez, 2008):

1. By inactivating the generated ROS at molecular level, thereby terminating the radical chain reaction (chain breaking).
2. By chelating the lead ion and preventing further formation of ROS.
3. By chelating lead and maintaining it in a redox state, which leads to its incompetency to reduce molecular oxygen.

Antioxidants may be broadly grouped according to their mechanism of action: *primary or chain breaking antioxidants and secondary or preventive antioxidants.*

**Figure 4:**
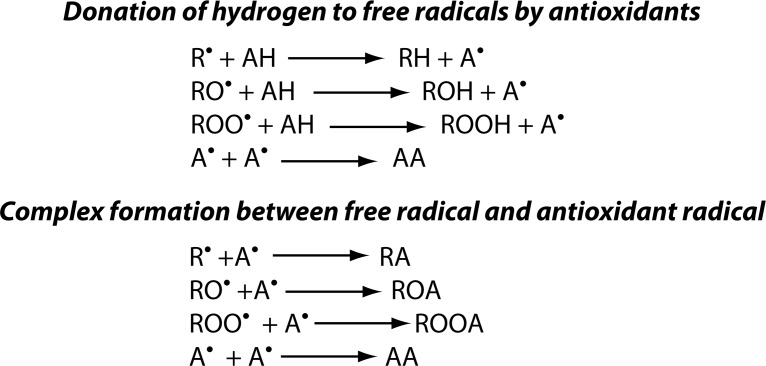
Chain breaking mechanism of antioxidants. R^•^, RO^•^, ROO^•^: Free Radicals, AH: Antioxidant, A^•^: Antioxidant radical

***Primary antioxidants*** are compounds capable of scavenging free radicals that are responsible for initiation or propagation of the chain reaction through *chain breaking mechanism* (Figure 4). This is done by donating free electrons to ROS and lipid radicals present in the biological system and converting them into stable molecules. This prevents or delays the oxidation process and prevents lipid peroxidation, which can cause membrane damage. Common primary antioxidants include flavonoids, tocopherol and ascorbic acid (Vaya & Aviram, 2000).

On the other hand, ***secondary antioxidants*** (like low molecular weight polyphenols) are those which mainly act by slowing down the rate of the oxidation reaction. The major difference between primary and secondary antioxidants is that the latter do not convert free radicals into stable molecules. They are capable of chelating heavy metals like lead (Wanasundara & Shahidi, 2005).

## Natural antioxidants and the present status

Naturally occurring antioxidants and their role in quenching free radicals generated in the body under various pathologic conditions have been an active area of research. Studies have revealed that antioxidants possess the ability of both preventing and curing the damage caused by the generation of free radicals in the body. Natural antioxidants can be categorized into *enzymatic* and *non enzymatic*.

**Figure 5:**
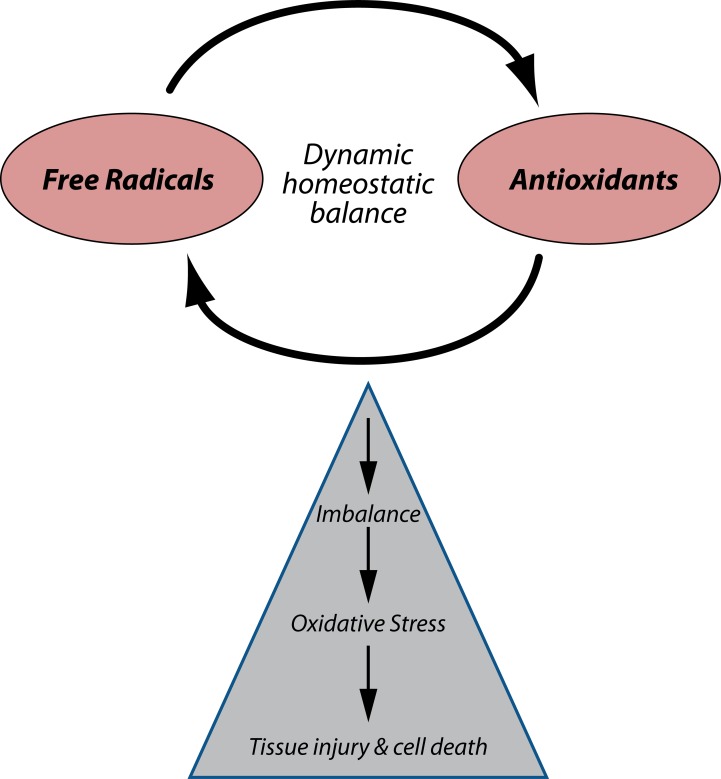
Under normal physiological conditions, there is a balance between free radicals and antioxidants and any deviation from it can cause oxidative stress leading to cell death.

Enzymatic antioxidants like SOD, CAT, GP_X_ are produced endogenously in the cells, whereas non enzymatic antioxidants like carotenoids, flavonoids, vitamins, minerals, *etc.* are constituents of many fruits, vegetables, nuts, grains and some meats (Flora, 2009). The amount of antioxidants present under normal physiological conditions is just adequate to quench the free radicals that are generated at a normal physiological rate. Any further increment in the concentration of free radicals (due to environmental or natural causes) can cause an imbalance between the free radicals and antioxidants, leading to oxidative stress (Blokhina *et al.*, 2003) (Figure 5). This is where the role of exogenous antioxidants becomes important. They are taken through the diet or in the form of supplements to maintain the homeostasis between free radicals and antioxidants and thus prevent various deleterious effects, like heavy metal toxicity, inflammation, cancer, aging, cardiovascular and brain disorders (Willcox *et al.,* 2004; Pietta, 2000).

It has been reported that those who take an antioxidant rich diet are at the forefront of reaping various health benefits. To boost antioxidant levels, food is always favored over supplements mainly because it contains thousands of antioxidants, in contrast to supplements, which are generally rich in a single or a few antioxidants.

This review will now incorporate a detailed study of some natural antioxidants that have been investigated and put forth for the treatment of lead induced oxidative stress.

## Vitamins

The role of vitamins (particularly B, C and E) has been found to be extremely significant and competitive in fighting toxicological manifestations of lead poisoning. These vitamins may chelate lead from the tissues along with restoring the pro/antioxidant balance. The role of various prominent vitamins in preventing lead toxicity has been discussed.

**Table 2:** Classification of natural antioxidants.

Enzymatic		Non-enzymatic				
Endogenous antioxidants (Cellular)	Phyto-antioxidants	Herbal antioxidants	Antioxidant vitamins	Antioxidant minerals	Antioxidant hormones	Thiol antioxidants
Catalase SOD GPX	Carotenoids β -carotene, Lycopene Lutein Flavonoids Quercetin Catechin Garlic Lipoic acid	Ginkgo Curcumin	Thiamine Pyridoxine Ascorbic acid α tocopherol	Selenium Zinc	Melatonin	Glutathione Thioredoxin

### Vitamin B (Pyridoxine and Thiamine)

Vitamin B6 (pyridoxine) and vitamin B1 (thiamine) are reported to have essential characteristics that can cure the deleterious effects of lead toxicity. Pyridoxine is an important co-factor which participates in the metabolic trans-sulfuration pathway which is responsible for the synthesis of cysteine from dietary methionine.

Vitamin B6 acts also as an antioxidant by stimulating the production of GSH and as a moderate chelator (Ahamed & Siddiqui, 2007). Chelation of lead by vitamin B6 could be attributed to the presence of the ring in the nitrogen atom or to the interference of vitamin B6 with the absorption of lead. Vitamin B1 (thiamine) has also been reported to exert protective efficacy against short-term implications of lead poisoning. Senapati *et al.* (2000) reported the protective role of thiamine hydrochloride on lead-induced endogenous lipid peroxidation in rat hepatic and renal tissues. They revealed a significant decrement in the levels of lead in liver and kidney.

### Ascorbic acid (vitamin C)

Ascorbic acid is probably the most widely studied vitamin when it comes to the prevention of lead induced oxidative stress. Its property of quenching ROS along with metal chelation makes it a potential detoxifying agent for lead (Das & Saha, 2010; Tariq, 2007). A recent study done by Chang *et al.* (2011) showed the defensive effect of ascorbic acid on oxidative stress, developed in the hippocampus of lead exposed suckling rats. They reported that introduction of ascorbic acid during pregnancy and lactation caused to some extent amelioration of oxidative stress in the developing hippocampus. Shan *et al.* (2009) examined the defensive effects of ascorbic acid and thiamine against the toxic effects of lead on testes of mice. Exposure to lead exhibited a significant decrease in epididymal sperm count and motility, along with the induction of apoptosis through activation of caspase-3, Fas/Fas-L and Bcl-2. Co-administration of ascorbic acid and thiamine reverted the oxidative stress in a concentration dependent manner, as well as DNA damage and apoptosis induced by lead in rat liver cells (Wang *et al.,* 2007). Supplementation of ascorbic acid in combination with silymarin was able to reduce acute hepatotoxic lead toxicity (Shalan *et al.,* 2005).

### Vitamin E (α-tocopherol)

Vitamin E is a fat soluble vitamin with numerous biological functions (Flora, 2002). It possesses powerful anti-oxidative properties, operative in the membrane to prevent lipid peroxidation by obstructing the free radical chain reaction. Sajitha *et al.,* (2010) reported that vitamin E administered to rats counteracted the deleterious effect of lead by scavenging free radicals and thus preventing oxidative stress. Lead induced ALAD inhibition in the erythrocytes was found to be reversed by the treatment with vitamin E (Rendon-Ramirez *et al.,* 2007). Vitamin E was also found to be helpful in restoring thyroid dysfunction by maintaining the hepatic cell membrane architecture disrupted indirectly by lead induced lipid peroxidation. Effect of vitamin E in combination with other antioxidants has been found to be more pronounced than its individual administration. Flora *et al.* (2003) reported that co-administration of vitamin E with monoisoamyl derivative (MiADMSA), which is a thiol chelator, exerts an elevated recovery from lead burden in rats. Interestingly, α-tocopherol is capable of reducing ferric iron to ferrous iron (*i.e.* to act as a pro-oxidant). Moreover, the ability of α-tocopherol to act as a pro-oxidant (reducing agent) or antioxidant depends on whether all of the α-tocopherol becomes consumed in the conversion from ferric to ferrous iron or whether, following this interaction, residual α-tocopherol is available to scavenge the resultant ROS (Yamamoto & Nike, 1988).

## Flavonoids

Flavonoids are naturally occurring polyphenolic compounds. They are the main constituents of fruits, vegetables and certain beverages (Youdim *et al.,* 2002). The anti-oxidative nature of flavonoids has been extensively investigated. These compounds, like other anti-oxidants, can cure or prevent oxidative stress by chelating redox active metal ions and also by terminating the free radical chain reaction (Terao, 2009; Rice-Evans, 2001).

**Figure 6:**
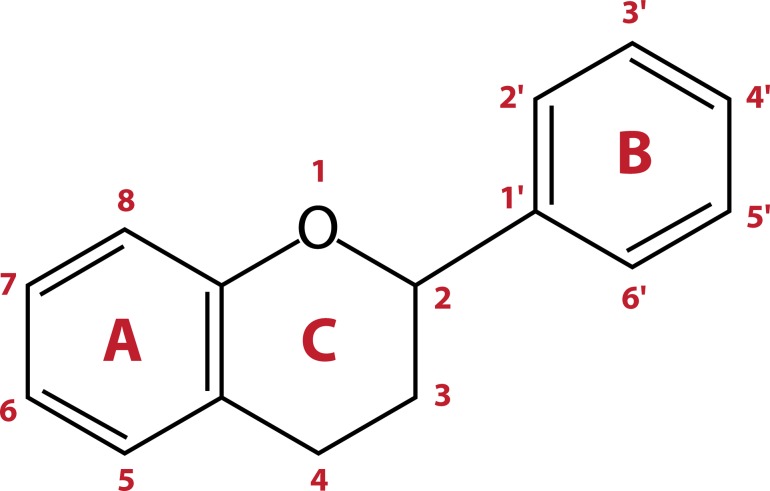
General structure of flavonoids.

The capacity of flavonoids to act as antioxidants depends upon their molecular structure (Figure 6). Their general structure includes a *diphenylpropane moiety* composed of two or more aromatic rings (A and B), each having at least one aromatic hydroxyl group connected via a carbon chain. The chain consists of three carbons that combine with an oxygen and two carbons of one of the aromatic rings (A ring) to form a third 6-member ring (C ring) known as the pyran ring (Larson *et al.* 2012).

The metal chelating ability of flavonoids arises from the appropriate positioning of the functional groups that include both the hydroxyl groups of ring-B and the 5-hydroxy group of ring-A. The electron donating capability of flavonoid molecules to scavenge ROS rests with the 3’,4’-catechol (dihydroxy) structure (B-ring). Another structural feature that contributes to the anti-oxidative nature is the presence of 2, 3 double bond in conjugation with a 4-oxo group in the C-ring (Heim *et al.,* 2002; Ng *et al.,* 2000; Dugas *et al.,* 2000).

### Quercetin

**Figure 7:**
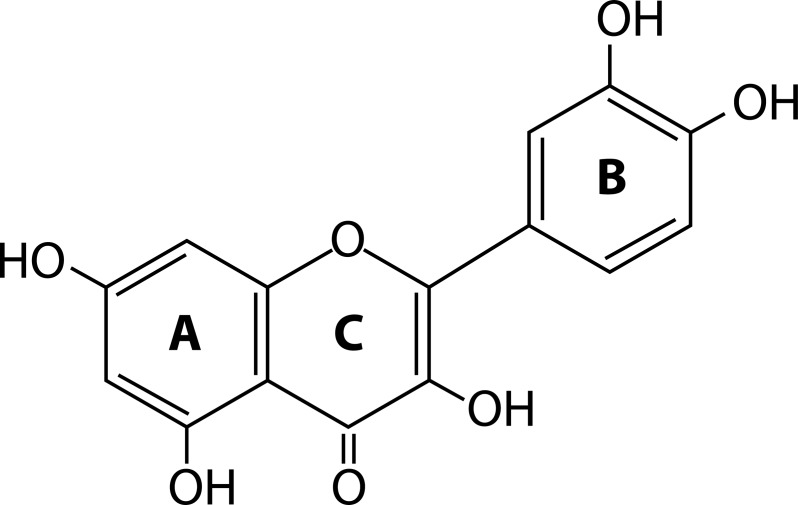
General structure of quercetin.

Quercetin is a ubiquitously distributed and comprehensively explored bioflavonoid. Dietary sources of quercetin include fruits, vegetables and tea. The chemical name for quercetin is 3,3’,4’,5,7-pentahydroxyflavone. The presence of multiple hydroxyl groups in its chemical structure and conjugated electrons account for its antioxidant and metal chelating property (Figure 7).

**Figure 8:**
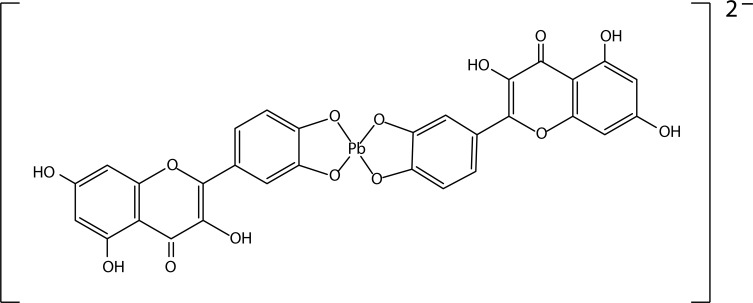
Structure of quercetin - Pb complex.

These hydroxyl groups along with the carbonyl group easily donate electrons by undergoing resonance and stabilize free radicals that can initiate lipid peroxidation (Beecher, 2003). Quercetin chelates lead by forming a coordination bond with the lead ions through its ortho-phenolic groups located on the quercetin B ring (Bravo & Anacona, 2001) (Figure 8). Liu *et al.* (2010) confirmed the protective role of quercetin on various lead induced histopathological injuries in the rat kidney. They reported that quercetin markedly decreased the ROS level and lowered the GSH/GSSG ratio in the kidney of lead treated rats. It also suppressed the increased level of 8-hydroxydeoxyguanosine, along with the restoration of Cu/Zn-SOD, CAT and GPx activities in the kidney of lead treated rats. TUNEL assay confirmed the inhibition of lead induced apoptosis in the rat kidney.

Hu *et al.* (2008) investigated the role of lead in inducing impairment of synaptic plasticity in the rat model and curing the same by administering quercetin. Various parameters were considered by the authors, like input/output (I/O) functions, paired-pulse reactions (PPR), excitatory postsynaptic potential (EPSP) and population spike (PS) amplitude in the dentate gyrus area of different lead treated rat groups. All of these showed significant improvement after treatment with quercetin. Reduction in hippocampal lead concentration was also reported. Thus, the medicinal and therapeutic properties of quercetin, along with its low toxicological profile, has made it a very promising drug in the field of heavy metal toxicity.

### Alpha Lipoic Acid

Alpha lipoic acid is an antioxidant synthesized in small amounts in the human body. It is also present in certain foods, including carrots, beets, spinach, potatoes and red meat (Durrani *et al.,* 2010). It is considered a *“universal antioxidant”* because it is both fat soluble and water soluble, enabling it to function in both fatty and aqueous regions of the body (De Araujo *et al.,* 2011). Its antioxidant activity tends to act in dual ways: first it attacks ROS and prevents the formation of lipid peroxides, and second, it can replenish and regenerate other antioxidants like vitamin C and E (Haleagrahara *et al.*, 2011). Lipoic acid has mostly been used in combination with other chelating agents like 2,3-dimercaptosuccinic acid (DMSA), due to the fact that lipoic acid itself does not have metal chelating ability but it can consistently tackle the generated oxidative stress. Sivaprasad *et al.* (2002, 2003 2004) explored the histopathological implications of lead exposure on kidneys, liver and erythrocyte membranes and showed that lipoic acid greatly improved the condition by reversing the developed oxidative stress. Lipoic acid was found to be more effective in removing lead from the brain compared to any other organs (liver, kidneys and other soft tissues) (Pande and Flora., 2002).

## Herbal antioxidants

The ability of herbal antioxidants to act as useful clinical medicine is due to their low cost and few side effects. However, actual implementation of herbal antioxidants as potential medicines has been highly limited. This is due to the longer treatment durations associated with it, which makes it a preventive rather than therapeutic measure. Apart from this, herbal drugs also suffer from a serious drawback of poor bioavailability in the body and require much higher and repetitive doses to maintain the therapeutic threshold in the body. A few herbal antioxidants that have been reported to provide protection against lead induced oxidative stress will be discussed.

### Garlic

Garlic is a medicinal plant that has been an inseparable part of Indian culinary for over 5000 years. Besides its use as a condiment, it is credited to have remarkable therapeutic and pharamcological properties. Its active agent is allicin, which imparts its characteristic odor as well as medicinal properties (Sharma *et al.*, 2010). Garlic can prevent oxidative stress by chelating lead ions and scavenging free radicals. Senapati *et al.* (2001) reported the prophylactic efficacy of garlic extract in reducing the lead burden from soft tissues. In another study, Pourjafar *et al.* (2007) further confirmed the ability of garlic to reduce the lead burden from the liver, kidney, blood and bone. The protective efficacy of aqueous garlic extract was studied against lead induced hepatic injury in rats. The results clearly indicated the ameliorative ability of garlic towards hepatic injury caused by lead due to generated oxidative stress (Kilikdar *et al.,* 2011).

### Curcumin

Curcumin is a yellow-colored polyphenolic compound and the principal active component of turmeric, which is obtained from the plant *Curcuma longa*. There are reports of antioxidant, radical scavenging and metal chelating effects of curcumin in metal toxicity (Sethi *et al.,* 2009; Agarwal *et al.,* 2010; Singh *et al.,* 2010; Rao *et al.,* 2008). Shukla *et al.* (2003) reported for the first time the protective effect of curcumin against lead-induced neurotoxicity in rats by showing significant improvement in the levels of various biomarkers of oxidative stress (GSH, SOD and CAT levels) in different regions of the brain. Daniel *et al.* (2004) provided insight into the chelating properties of curcumin by showing a remarkable reduction in levels of lead in rat brains. The above mentioned results on curative effects of curcumin on lead neurotoxicity were successfully reproduced by Dairam *et al.* (2007) in male rats. In spite of these commendable properties, the major drawback associated with the use of curcumin is its low bioavailability. This is due to its poor aqueous dispersion and poor absorption from the intestine coupled with a high degree of metabolism of curcumin in the liver and rapid elimination in bile (Maiti *et al.,* 2007).

#### Centella asiatica

*Centella asiatica,* popularly known as *Indian Pennywort,* is well known for its various medicinal applications and for treating metal toxicity (Flora & Gupta, 2007)**.** Introduction of C. *asiatica* along with chelating agents like DMSA have shown very promising results regarding treatment of lead induced oxidative stress in rats (Saxena & Flora, 2006). Independent administration of *C. asiatica* has also been studied regarding its ameliorative ability against lead induced oxidative stress. The results found were quite encouraging (Ponnusamy *et al.,* 2008; Sainath *et al.,* 2011). It has been hypothesized that *C. asiatica* can cross the BBB and restore the levels of altered neurotransmitters and can also restore the impaired prooxidant/antioxidant balance arising due to lead exposure (Hussin *et al.*, 2007).

## Recent Strategies

The major drawback in the usefulness of antioxidants is their poor bioavailability due to low solubility and rapid clearance. Although the pharmacological safety of most of the therapeutics promises a great potential for treatment and prevention of various diseases, their relatively low bioavailability is a major hurdle for clinical development. Novel approaches to overcome the problem of low bioavailability of these antioxidants are being developed. These approaches include improved formulations for better delivery such as liposomes, micelles, phospholipid complexes and nanoparticles (Anand *et al.,* 2007).

### Liposomal Nanoparticles

Lipid-based nanoencapsulation systems enhance the performance of antioxidants by improving their solubility, bioavailability and by preventing unwanted interactions with other food components. The main lipid-based nanoencapsulation systems that can be used for the delivery of nutraceuticals are nanoliposomes, nanocochleates and archaeosomes (Mozafari *et al.,* 2008). Nanoliposome technology presents exciting opportunities for food technologists in areas such as encapsulation and controlled release of antioxidants, as well as the enhanced bioavailability, stability and shelf-life of sensitive ingredients. Application of nanoliposomes as carrier vehicles of nutrients, nutraceuticals, enzymes, food additives and food antimicrobials was reported (Mozafari *et al.,* 2006). Liposomes are phospholipid bilayers that have closed in upon themselves to form a tiny bubble or vesicle. These nanoparticles have hydrophilic heads pointing outward that make them water soluble, while the hydrophobic tails of both lipid layers interact. The inside of the liposome is also water soluble, enabling it to protect soluble drugs, cosmetics or biomolecules intended for delivery into cells. The outer or inner membrane structures can be altered with charged ligands or target-specific molecules, such as antigens, for specialized use. Encapsulation of curcumin in liposomes with their hydrophilic and hydrophobic properties should offer an excellent antioxidant potential. While these studies suggest that liposomal curcumin is bioactive, the increased bioavailability of encapsulated curcumin *in vivo* still needs to be conclusively demonstrated (Gandhi *et al.*, 2011).

### Nanoencapsulation

Nanoencapsulation of antioxidants provides improved biodistribution and bioavailability of poorly-soluble therapeutics through solubilization. Many vehicles have been developed for encapsulation and delivery of therapeutics, including solid nanoparticles, micelles, lipid polymer vesicles (polymersomes) and nanohydrogels. In recent years, biodegradable polymeric nanoparticles have attracted considerable attention. In spite of the development of various synthetic and semi-synthetic polymers, natural polymers are still widely used. Some of them are: gums (acacia, guar, *etc*.), chitosan, gelatin, sodium alginate, albumin *etc*. Polymeric nanoparticles for controlled release and targeted delivery of functional compounds have been reported in the literature (Zigoneanu *et al.*, 2008). They are synthesized using polymers and surfactants and include alginic acid, polylactic-co-glycolic acid and chitosan. A recent study using a polymer-based nanoparticle of curcumin found that its molecular activity was similar to free curcumin in a pancreatic cell line (Bisht *et al.,* 2007). Encapsulation of curcumin in a pluronic block copolymer demonstrated a slow and sustained release of curcumin and showed anticancer activity comparable with free curcumin (Sahu *et al.,* 2010). Properties such as biodegradability, low toxicity and good biocompatibility make nanoparticles suitable for use in biomedical and pharmaceutical manipulations. Thus, nanotechnology-based approaches to increase curcumin delivery, bioavailability and high therapeutic potential *in vivo* are gradually evolving (Choudhuri *et al.,* 2005). There are several lines of evidence from *in vitro*, *in vivo*, preclinical and clinical studies to suggest that curcumin has a great potential in the prevention and treatment of various diseases, yet the potential role of nanoencapsulated curcumin has not been thoroughly explored. In the near future, enhanced bioavailability of curcumin by nanoencapsulation is likely to bring this promising natural product to the forefront of therapeutic agents for treatment of human diseases.

## Conclusion

Lead poisoning has been known to mankind since antiquity, although the situation got aggravated since the 18^th^ century during the industrial revolution. It was the period when various important qualities of lead were discovered that made it one of the most widely used industrial metals. Lead has no known biological function in the body and once it enters the body, it is known to cause severe health effects that might be irreversible. It affects almost all the major organ systems of the body like hematopoietic, renal, nervous and cardiovascular systems. Various molecular, cellular and intracellular mechanisms have been proposed to explain the toxicological profile of lead that includes generation of oxidative stress, ionic mechanism and apoptosis. Of these oxidative stress has been found to be more pronounced and much more severe. Lead causes generation of ROS which results in critical damage to various biomolecules like DNA, enzymes, proteins and membrane based lipids, while simultaneously it impairs the antioxidant defense system. Chelation therapy has so far been used as the mainstay of the treatment that involves quenching of lead from different sites of the body and expels it through urine. Prevention is regarded as the best approach, involving incorporation of various natural and synthetic antioxidants. Various naturally occurring antioxidants (nutrient antioxidants) like vitamins, flavonoids and herbal antioxidants have been reported for the prevention and treatment of lead induced toxicity and oxidative stress in particular. They have the ability to scavange ROS at molecular level and chelate lead ions, thereby reversing the toxic effects. These antioxidants were also reported to provide an elevated therapeutic impact when administered with chelating agents like DMSA, which is a thiol chelator. Nevertheless, we do recommend that the presence and possible beneficial effects of antagonists be carefully considered, as an antioxidant may become a pro-oxidant in the presence of certain other molecules. For example, chlorophylls may overwhelm the antioxidant effect of phenolics due to photosensitized oxidation, while transition metal ions, as those of iron and copper, may render conditions favoring oxidation. Synergism among different phenolic antioxidants and between phenolics and non-phenolics should also be considered in all application areas.

The latest addition to preventive regimens is the use of nanoencapsulation or liposome mediated drug delivery, which deals with the problem of low systemic bioavailability of certain natural hydrophobic antioxidants, like curcumin. Compared to conventional drugs, this approach also reduces the dosage needed to maintain its therapeutic threshold in the body.
